# Inhibition of Monoacylglycerol Lipase Decreases Angiogenic Features of Endothelial Cells via Release of Tissue Inhibitor of Metalloproteinase-1 from Lung Cancer Cells

**DOI:** 10.3390/cells12131757

**Published:** 2023-06-30

**Authors:** Felix Wittig, Lino Henkel, Jan Lukas Prüser, Jutta Merkord, Robert Ramer, Burkhard Hinz

**Affiliations:** Institute of Pharmacology and Toxicology, Rostock University Medical Center, Schillingallee 70, 18057 Rostock, Germany; felix.wittig@med.uni-rostock.de (F.W.); lino.henkel@freenet.de (L.H.); lukas_prueser@web.de (J.L.P.); jutta.merkord@t-online.de (J.M.); robert.ramer@med.uni-rostock.de (R.R.)

**Keywords:** angiogenesis, monoacylglycerol lipase, JZL184, TIMP-1, lung cancer cells

## Abstract

Despite the well-described anticarcinogenic effects of endocannabinoids, the influence of the endocannabinoid system on tumor angiogenesis is still debated. In the present study, conditioned medium (CM) from A549 and H358 lung cancer cells treated with ascending concentrations of the monoacylglycerol lipase (MAGL) inhibitor JZL184 and 2-arachidonoylglycerol (2-AG), a prominent MAGL substrate, caused a concentration-dependent reduction in human umbilical vein endothelial cell (HUVEC) migration and tube formation compared with CM from vehicle-treated cancer cells. Comparative experiments with MAGL inhibitors JW651 and MJN110 showed the same results. On the other hand, the angiogenic properties of HUVECs were not significantly altered by direct stimulation with JZL184 or 2-AG or by exposure to CM of JZL184- or 2-AG-treated non-cancerous bronchial epithelial cells (BEAS-2B). Inhibition of HUVEC migration and tube formation by CM of JZL184- and 2-AG-treated A549 cells was abolished in the presence of the CB_1_ antagonist AM-251. Increased release of tissue inhibitor of metalloproteinase-1 (TIMP-1) from JZL184- or 2-AG-stimulated A549 or H358 cells was shown to exert an antiangiogenic effect on HUVECs, as confirmed by siRNA experiments. In addition, JZL184 caused a dose-dependent regression of A549 tumor xenografts in athymic nude mice, which was associated with a decreased number of CD31-positive cells and upregulation of TIMP-1-positive cells in xenograft tissue. In conclusion, our data suggest that elevation of 2-AG by MAGL inhibition leads to increased release of TIMP-1 from lung cancer cells, which mediates an antiangiogenic effect on endothelial cells.

## 1. Introduction

Tumor neovascularization or tumor angiogenesis is considered a hallmark of cancer that allows solid tumors to grow beyond 2–3 mm in diameter [1]. To maintain vascular supply during rapid growth, malignant tumors secrete pro-angiogenic factors such as vascular endothelial growth factor (VEGF) in response to hypoxia and malnutrition, which chemoattract vascular cells and promote neovascularization [2]. This “angiogenic switch” involves several processes, including local matrix degradation, migration of endothelial cells into tumor tissue, and morphological changes in endothelial cells to form tubular structures. The steps of tumor neovascularization are supported by matrix metalloproteinases (MMPs), a group of zinc-dependent endopeptidases released from cancer tissue into the tumor microenvironment [3,4]. In this context, the endogenous tissue inhibitor of metalloproteinase-1 (TIMP-1) was shown to suppress tumor neovascularization and the angiogenic capacity of endothelial cells [5,6,7,8,9]. Accordingly, in a previous study conducted by us, recombinant TIMP-1 was found to reduce migration as well as tube and three-dimensional sprout formation of HUVECs, while viability remained virtually unchanged [6].

In addition to clinically approved antiangiogenic drugs such as bevacizumab, sunitinib, sorafenib, pazopanib (for review, see [10]), and aflibercept [11], cannabinoid compounds attracted scientific interest due to their antiangiogenic properties. Thus, various cannabinoids of plant or synthetic origin were repeatedly reported to induce tumor regression associated with reduced angiogenesis in experimental tumors in glioma [12], skin [13], colon [14], and lung cancer models [15,16]. A number of cannabinoids thereby directly inhibit the angiogenic properties of vascular endothelial cells [12,14] and reduce the expression of pro-angiogenic factors such as VEGF [12,17] and angiopoietin-2 [12] in tumor cells.

On the other hand, there is limited evidence on the influence of endocannabinoid degradation inhibitors on tumor angiogenesis. Of particular interest as a target herein is the enzyme monoacylglycerol lipase (MAGL), which is responsible for the degradation of the endocannabinoid 2-arachidonoylglycerol (2-AG) [18]. Corresponding enzyme inhibition leads to an increase in antitumor-acting 2-AG and decreased synthesis of protumorigenic fatty acids (for review, see [19,20]). In fact, a number of studies showed that inhibition or knockdown of MAGL mediates tumor-regressive [21,22], anti-invasive [21,22,23,24,25,26], and antimetastatic effects [23,26,27,28]. These preclinically demonstrated anticancer effects also appear promising in light of the analgesic effects shown for MAGL inhibitors, which, according to a recent preclinical study, are also associated with complete inhibition of chemotherapy-induced neuropathy [29]. The hitherto rarely published associations of low or high MAGL expression in different tumor types with patient outcomes were recently reviewed [20].

However, in the context of MAGL inhibition as a potential anticancer strategy, there are limited data on antiangiogenic effects in the literature, which also never adequately addressed the role of 2-AG in this process [30]. Moreover, both in the case of 2-AG and in corresponding pharmacological strategies to increase this endocannabinoid and MAGL substrate, a possible interference in the communication between tumor and endothelium, which is important for antiangiogenesis, so far remained unnoticed. The present study therefore sought to elucidate the effect of MAGL inhibition on angiogenic crosstalk between cancer and endothelial cells. Most experiments were performed with JZL184, a piperidine carbamate that irreversibly and selectively inhibits MAGL by carbamoylation of the active site catalytic serine nucleophile [31]. Considering the aforementioned antiangiogenic effect of TIMP-1 [6] and the recent demonstration of TIMP-1 induction by MAGL inhibitors in relation to their anti-invasive properties [23], a possible role of this glycoprotein was the focus of further mechanistic investigations. Here, we demonstrate for the first time an indirect antiangiogenic effect of a MAGL inhibitor and the MAGL substrate 2-AG on endothelial cells mediated via CB_1_ receptors and increased release of TIMP-1 from cancer cells. Consistent with this, JZL184-mediated dose-dependent tumor regression in A549 xenograft athymic nude mice was associated with decreased numbers of CD31-positive cells and upregulation of TIMP-1-positive cells in xenograft tissue. These results define MAGL as a remarkable target for innovative antiangiogenic cancer treatment options and also provide new insights into the role of the endocannabinoid system as an endogenous anticancer system.

## 2. Materials and Methods

### 2.1. Materials

JZL184, 2-AG, AM-251, AM-630, and palmitic acid were from Cayman Chemical (Ann Arbour, USA). RHC 80267 was from Merck (Darmstadt, Germany). Dimethyl sulfoxide (DMSO), ethylenediaminetetraacetic acid (EDTA), glycerol, glycine, sodium chloride (NaCl), Tris hydrocloride (Tris-HCl), and Tris ultrapure were purchased from AppliChem (Darmstadt, Germany). 4-(2-hydroxyethyl)-1-piperazineethanesulfonic acid (HEPES) and β-mercaptoethanol were bought from Ferak (Berlin, Germany). *Aqua ad iniectabilia* was bought from Braun Melsungen AG (Melsungen, Germany). JW651, MJN110, capsazepine, aprotinin, bovine serum albumin, HCl, hydrogen peroxide solution (H_2_O_2_), luminol, bromophenol blue, orthovanadate, *p*-coumaric acid, paraformaldehyde, phenylmethylsulfonyl fluoride (PMSF), and Triton^®^ X-100 were from Sigma-Aldrich (Taufkirchen, Germany). Leupeptin was purchased from Biomol (Hamburg, Germany). Acrylamide (Rotiphorese^®^ Gel 30), ammonium peroxydisulphate (APS), *N*,*N*,*N′*,*N′*-Tetramethylethylenediamine (TEMED), and Tween^®^ 20 were obtained from Carl Roth GmbH (Karlsruhe, Germany). Methanol was bought from J. T. Baker (Griesheim, Germany). Dulbecco’s Modified Eagle Medium (DMEM) with 4.5 g/L glucose and with UltraGlutamine^TM^ I was from Lonza Cologne GmbH (Cologne, Germany). Phosphate-buffered saline (PBS) and fetal calf serum (FCS) were bought from PAN Biotech (Aidenbach, Germany). Milk powder was obtained from Bio-Rad Laboratories GmbH (Munich, Germany). Gibco^TM^ Penicillin-Streptomycin (10,000 U/mL), Gibco^TM^ Trypsin-EDTA, Gibco^TM^ Trypan Blue Solution, Lipofectamine™ RNAiMAX Transfection Reagent, and OptiMEM™ Reduced-Serum Medium were from Thermo Fisher Scientific Inc. (Schwerte, Germany).

### 2.2. Cell Culture

A549 human lung carcinoma cells were purchased from DSMZ (Deutsche Sammlung von Mikroorganismen und Zellkulturen GmbH, Braunschweig, Germany; DSMZ cat. No. ACC-107, RRID:CVCL_0023). Species confirmation as human was carried out by the supplier using isoelectric focusing of malate dehydrogenase, nucleosid phosphorylase, and fingerprint. Multiplex PCR of minisatellite markers revealed a unique DNA profile. NCI-H358 human lung carcinoma cells (ATCC: CRL-5807™; RRID:CVCL_1559; further referred to as H358 cells) and the human bronchial epithelial cell line BEAS-2B (ATCC: CRL-9609™; RRID:CVCL_0168) were obtained from ATCC-LGC (Wesel, Germany). Cell line confirmation of H358 and BEAS-2B were carried out by the supplier using cytogenetic analyses. A549 and H358 lung carcinoma cells, as well as BEAS-2B cells, were cultured in DMEM supplemented with 10% heat-inactivated FCS, 100 U/mL penicillin, and 100 μg/mL streptomycin. All incubations of these cells were performed in serum-free DMEM. HUVECs were obtained from PromoCell as cryopreserved single-donor samples (Heidelberg, Germany; #C-12200; RRID:CVCL_2959) and cultivated using the Endothelial Cell Growth Medium Kit (#C-22110) from the same company. For experiments with HUVECs, cells were used between passages 2 and 7. All cell lines were frozen in large stock at early passages and were used within 6 months following resuscitation. 

Test substances were dissolved in DMSO (JZL184, 2-AG, JW651, MJN110, AM-251, AM-630, capsazepine, and RHC 80267) or ethanol (palmitic acid), after which the stock solutions were diluted with PBS. The final solvent concentrations per substance used in the cell incubates were 0.1% (*v*/*v*) DMSO or 0.1% (*v*/*v*) ethanol. Even when several test substances were added to the cells, the final concentration of DMSO in the incubates did not exceed 0.3% (*v*/*v*) DMSO. The vehicle control contained the respective concentrations of DMSO and ethanol. In all experiments, the incubation media of the vehicle and substance-treated cells contained the same amount of solvent. The 2-AG was supplied by the producer as a mixture of 2-AG and 1-AG (9:1). Therefore, all stated concentrations in the Results section refer to nine parts 2-AG and one part 1-AG.

### 2.3. Treatment Protocol to Assess Direct Substance Effects on HUVEC Migration, Viability, and Tube Formation

To assess HUVEC viability after direct exposure to JZL184 and 2-AG, HUVECs were seeded at a density of 5 × 10^3^ cells per well in 96-well plates and were allowed to adhere for 24 h in endothelial cell growth medium. The cells were then washed and incubated with vehicle or test substances in serum-free DMEM for 24 h. 

To assess the direct effects of JZL184 and 2-AG on HUVEC migration and tube formation, the cells were suspended in serum-free DMEM containing the vehicle or the indicated concentrations of the respective test substance. HUVEC suspensions adjusted to a density of 1 × 10^5^ HUVECs (migration) or 5 × 10^4^ HUVECs (tube formation) were seeded into inserts (migration) or into the wells of a prepared 48-well plate (tube formation). Then, for migration analyses, DMEM was loaded into the lower companion plate with 10% FCS, which serves as a chemoattractant, and incubations were continued for another 24 h. Finally, the cells on the upper side of the inserts were removed with a cotton swab. To assess the direct effects of the test substances on tube formation, the closed intersections of HUVECs were counted in an investigator-blinded manner after a 2 h incubation period.

Migration, viability, and tube formation assays are described in detail in Section 2.5, Section 2.6 and Section 2.7.

### 2.4. Treatment Protocol to Assess Indirect Substance Effects on HUVEC Migration, Viability, and Tube Formation

For generation of CM from A549, H358, and BEAS-2B, cells were seeded at a density of 1 × 10^5^ cells per well of a 24-well plate (excepts for treatments of cells with JW651, MJN110, RHC 80267, and experiments with combinations of JZL184 and palmitic acid in which 5 × 10^4^ cells per well of a 48-well plate were used), grown to confluence, and treated for 48 h with the indicated test compounds in a final volume of 300 µL of serum-free DMEM. After incubation with vehicle or test compounds in serum-free DMEM, CM was collected and stored temporarily on ice. Meanwhile, HUVECs were washed, trypsinized, and counted. HUVECs were then resuspended in the respective CM. A total of 300 μL of the resulting HUVEC suspensions containing the pre-adjusted number of 1 × 10^5^ HUVECs were seeded onto the upper chamber of a modified Boyden chamber system for evaluation of migration. This involved a 24 h incubation with CM. For viability assays, 5 × 10^3^ HUVECs were seeded in 100 μL CM per well of a 96-well plate and incubated for 24 h before viability was measured by the WST-1 colorimetric assay. For tube formation assays, 5 × 10^4^ HUVECs suspended in 200 μL CM were seeded per well onto the Matrigel layers of a 48-well plate and then incubated for 2 h. 

Since serum-free medium without prior contact with cells does not contain components released by tumor cells, this reference group was omitted from experiments addressing the impact of test substances on TIMP-1 expression and the possible involvement of cannabinoid receptors, palmitic acid, and TIMP-1 in the modulation of angiogenic properties by JZL184 and 2-AG. Treatment variations in siRNA transfection experiments are indicated in the respective section (see Section 2.9).

Migration, viability, and tube formation assays are described in detail in Section 2.5, Section 2.6 and Section 2.7.

### 2.5. Cellular Viability Analysis

The cellular viability of HUVECs was determined with a colorimetric assay that detects the cleavage of WST-1 (Roche Diagnostics, Mannheim, Germany), a water-soluble tetrazolium salt (4-[3-(4-iodophenyl)-2-(4-nitrophenyl)-2H-5-tetrazolio]-1,3-benzenedisulfonate), to a soluble formazan dye by metabolically active cells. Cell metabolic activity was determined by measuring the absorbance at 450/690 nm with an ELISA plate reader.

### 2.6. Migration Assay

The effect of test compounds on HUVEC migration was determined by a modified Boyden chamber assay using Falcon^®^ cell culture inserts (8 µm pore size; Corning Inc., Corning, NY, USA). In this assay, cells seeded onto the inserts must migrate through a polyethylene terephthalate membrane with 8 µm pores to a chemoattractant in the 24-well companion plate (lower compartment) as previously described [6]. Briefly, after resuspension of 1 × 10^5^ HUVECs per sample in serum-free medium containing vehicle (unconditioned medium; UCM) or CM obtained from A549, H358, or BEAS-2B cells, HUVEC suspensions were seeded into the upper chambers. DMEM containing 10% FCS was added to the lower companion plate as a chemoattractant. Neither CM nor UCM in the upper Boyden chamber contained serum. After incubation in a humidified incubator at 37 °C and 5% CO_2_ for 24 h, the non-migrating HUVECs on the upper surface of the inserts were removed with a cotton swab and the viability of the migrated cells on the lower surface was quantified using the colorimetric WST-1 test.

### 2.7. Tube Formation Assay

To visualize and quantify the angiogenic potential of the test compounds or CM derived from A549, H358, or BEAS-2B cells on HUVECs, tube formation assays were performed on 48-well plates coated with Matrigel as previously described [5,6] with slight modifications. This assay is based on the finding that in vitro organization of endothelial cells into capillary-like networks on Matrigel layers mimics the cellular behavior of an angiogenic process in vivo [32]. To perform this assay, 48-well plates were coated with 30 µL of ice-cooled Corning^®^ Matrigel^®^ Matrix (BD Biosciences, Heidelberg, Germany; #356234, referred to as Matrigel) and polymerized at 37 °C for 2 h. Furthermore, HUVECs were resuspended in serum-free DMEM containing vehicle or CM of lung cancer cells or BEAS-2B cells when the effect of CM was analyzed, or in serum-free DMEM containing vehicle or test compounds when the direct effect of the latter was analyzed. Thereafter, HUVECs were seeded at a density of 5 × 10^4^ cells per well in 48-well plates coated with Matrigel for a 2 h incubation period. Tube formation was analyzed quantitatively in total microscopic fields by counting the numbers of tube-like structures that formed closed intersections in an investigator-blinded fashion.

### 2.8. Western Blot Analysis

For analysis of protein levels, A549 and H358 cells were seeded into 48-well plates at a density of 1 × 10^5^ cells per well in 10% FCS containing medium or additionally transfected according to the protocol mentioned under “siRNA transfection”. Cells were incubated with vehicle or test substances in serum-free DMEM. Following a 48 h incubation period, CM were collected, centrifuged at 1300× *g* for 5 min, and used for subsequent Western blot analyses of TIMP-1. The respective cell lysates were used for further analysis of β-actin. For analysis of β-actin, cells were lysed in solubilization buffer (50 mM HEPES pH 7.4, 150 mM NaCl, 1 mM EDTA, 1% [*v*/*v*] Triton^®^ X-100, 10% [*v*/*v*] glycerol, 1 mM PMSF, 1 mM orthovanadate, 1 μg/mL leupeptin, and 10 μg/mL aprotinin) and centrifuged at 20,817× *g* for 5 min. Supernatants were used for Western blot analysis. Total protein amounts of cell lysates were determined using the bicinchoninic acid assay (Pierce, Rockford, IL, USA). Equal amounts of protein from cell lysates (β-actin) and equal volumes of CM (TIMP-1) were separated using 10% sodium dodecyl sulfate-polyacrylamide gels and subsequently transferred to nitrocellulose membranes, which were then blocked in 5% blotting grade blocker. Due to the lack of a housekeeping protein secreted into CM, TIMP-1 Western blot analysis of CM was supplemented with β-actin analysis of the respective cell lysates to monitor the possible toxic effects of the test substances. Blots were probed with primary antibodies raised against β-actin (Sigma-Aldrich, #A5316, RRID:AB_476743) or TIMP-1 (Merck Millipore, Darmstadt, Germany; #MAB3300, RRID:AB_2204544). Subsequently, membranes were washed and probed with a horseradish peroxidase-conjugated Fab-specific anti-mouse IgG (#7076, RRID:AB_330924) from Cell Signaling Technology Europe (Leiden, The Netherlands). Antibody binding was visualized using a chemiluminescence solution (100 mM Tris hydrochloride pH 8.5, 1.25 mM luminol, 200 µM *p*-coumaric acid, 0.09% [*v*/*v*] H_2_O_2_). The densitometric analysis of the band intensities was achieved by optical scanning and quantification with the Quantity One 1-D analysis software (Bio-Rad Laboratories GmbH, Munich, Germany). A pre-colored SDS-PAGE standard (Broad Range; Bio-Rad, Munich, Germany) was used to identify the band sizes. For the evaluation of changes in protein expression, the vehicle controls were defined as 100%.

### 2.9. SiRNA Transfections

SiRNA transfection was performed using the Lipofectamine™ RNAiMAX Reagent according to the manufacturer’s manual indicated under “Reverse Transfection” with slight modifications. RNAi-Lipofectamine™ RNAiMAX complexes were prepared in Opti-MEM^®^ I Reduced-Serum Medium containing 0.2 μL Lipofectamine™ RNAiMAX per 100 μL. Subsequently, RNA suspension buffers without siRNA, with TIMP-1 siRNA (Qiagen, Hilden, Germany; #1027418, SI00745318), or with a non-silencing control sequence siRNA (nonsi; Qiagen, Hilden, Germany; #1022076) were added. Dilutions were mixed, 100 μL was preloaded into each well of a 48-well plate, and incubated for 10–20 min. Finally, 1 × 10^5^ A549 or H358 cells suspended in a volume of 500 μL DMEM containing 10% FCS were added to each well, yielding a final concentration of 0.2 nM TIMP-1 siRNA or non-silencing siRNA, respectively. Following a 24 h incubation period, cells were washed and treated with vehicle, JZL184 or 2-AG in 300 μL serum-free DMEM. After a further 48 h, CM were collected, centrifuged at 1300× *g* for 5 min, and used to either prepare HUVEC suspensions for subsequent assays or to perform Western blot analysis of TIMP-1. For analysis of β-actin, cell lysates were used according to the protocol indicated under “Western Blot Analysis”.

### 2.10. Animal Experiments 

Tumors were induced in female nude mice (NMRI-nu/nu) by subcutaneous inoculation of 1 × 10^7^ A549 cells into the right dorsal flank. Animals were injected intraperitoneally every 72 h with JZL184 or its vehicle for 28 days. The treatment was started 7 days after tumor induction. Tumor volume was calculated as (4π/3) × (width/2)^2^ × (length/2). Experiments were conducted in accordance with the policies of the local Animal Ethics Committee. The mice were provided by the Animal Core Facility of the Rostock University Medical Center and were kept under specified pathogen-free conditions.

### 2.11. Immunhistochemical Analysis of CD31 and TIMP-1

The primary antibody against CD31 (angiogenesis marker) was purchased from BD Biosciences (#550274, RRID:AB_393571). The secondary antibody for CD31 was a polyclonal biotin-labeled goat anti-rat IgG (BD Biosciences). Primary TIMP-1 antibody was purchased from R&D Systems (Wiesbaden-Nordenstadt, Germany; #AF970, RRID:AB_355751). The secondary antibody for TIMP-1 was a polyclonal biotin-labeled goat anti-rat IgG (BD Biosciences: Goat IgG VisUCyte HRP Polymer; #VC004, RRID not yet available).

Visualization of antibody binding was conducted using the DAB Substrate Kit (BD Biosciences) as chromogens. Quantitative evaluation was conducted by counting strongly brown-stained cells (CD31- or TIMP-1-positive) in microscopic views in an investigator-blinded fashion. For statistical analyses, the percentage of positive cells among the respective total cell number per visual field was used to indicate the mean percentage of CD31- or TIMP-1-positive cells per tumor section.

### 2.12. Statistics

Comparisons between two groups were carried out using Student’s unpaired two-tailed *t* test. Comparisons between more than two groups were performed by one-way ANOVA with Bonferroni’s or Dunnett’s post hoc test. In the case of Bonferroni’s post hoc test, the determination of statistical significance was limited to the groups of interest for reasons of clarity of presentation. All statistical analyses were conducted with GraphPad Prism 7.02 (GraphPad Software, Inc., San Diego, CA, USA).

## 3. Results

### 3.1. JZL-184 and 2-AG Do Not Inhibit the Angiogenic Capacity of HUVECs When Administered Directly to Endothelial Cells, but When CM Is Used from A549 Cells Previously Treated with JZL-184 or 2-AG

**Figure 1:**
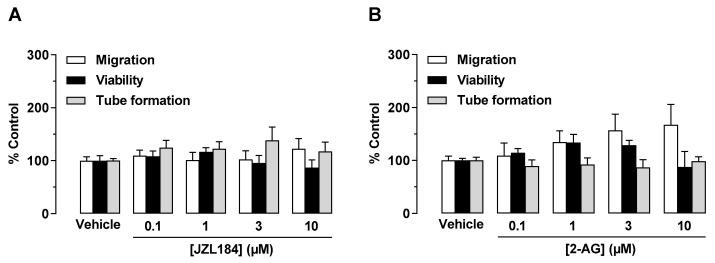
Direct effect of JZL184 (**A**) and 2-AG (**B**) on migration, viability, and tube formation of HUVECs. Migration (Boyden chamber assays, white bars), viability (WST-1 assay, black bars), and tube formation (tube formation assays, gray bars) of HUVECs after incubation with JZL184 (**A**) or 2-AG (**B**). The incubation time of HUVECs was 24 h for the migration and viability assay and 2 h for the tube formation assay. All percentage values given refer to the respective vehicle control set to 100%. Data represent mean ± SEM of *n* = 6 per group. Statistically significant differences between vehicle- and test substance-treated groups were excluded using one-way ANOVA with Dunnett post hoc test.

To determine the mechanism underlying a potential antiangiogenic effect of JZL184 and 2-AG, different angiogenic capacities of endothelial cells were first investigated in vitro. To this end, HUVECs were directly treated with JZL184 or 2-AG, followed by studies on migration, tube formation, and viability, as previously described [6]. According to Figure 1, it was found that neither JZL184 (Figure 1A) nor 2-AG (Figure 1B) significantly affected HUVEC migration, viability, and tube formation. Rather, in the case of 2-AG (Figure 1B), a stimulation of migration was detected, although this effect was not significant due to higher variance.

**Figure 2:**
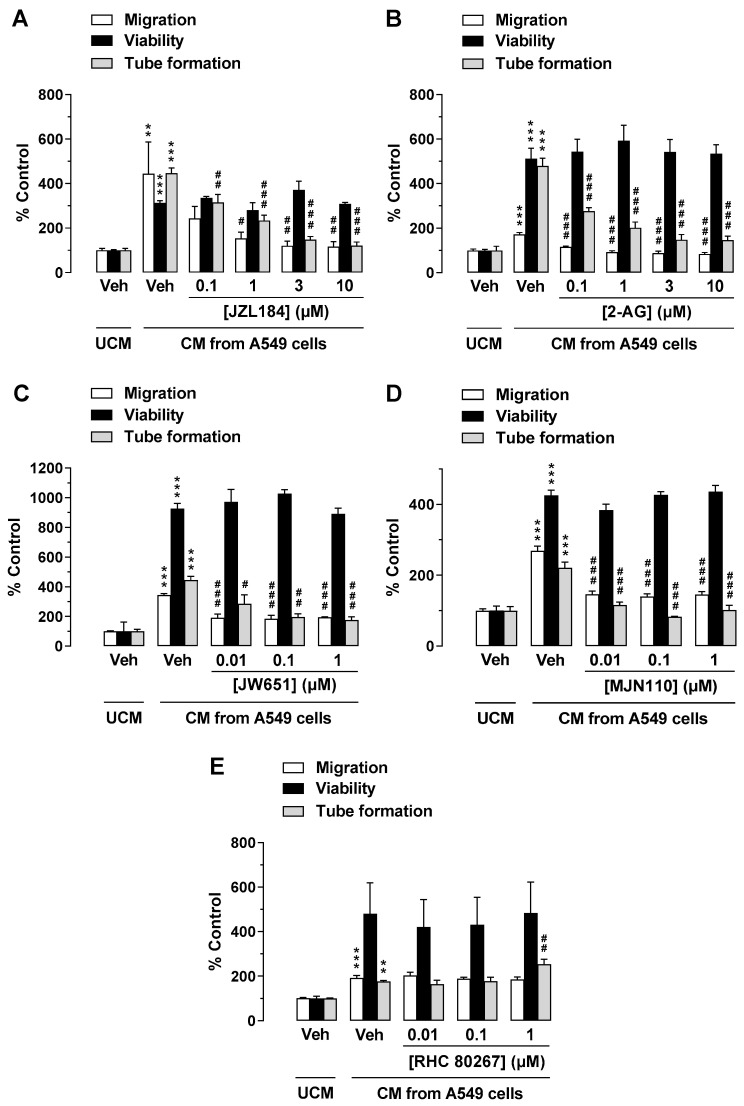
Effects of conditioned medium (CM) obtained from A549 cells treated with MAGL inhibitor JZL184 (**A**), MAGL substrate 2-AG (**B**), additional MAGL inhibitors JW651 (**C**) and MJN110 (**D**), or DAGL inhibitor RHC 80267 (**E**) on migration, viability, and tube formation of HUVECs. Migration (Boyden chamber assay, white bars), viability (WST-1 assay, black bars), and tube formation (tube formation assays, gray bars) of HUVECs were determined after incubation with CM from A549 cells for 24 h (migration and viability assay) or 2 h (tube formation analysis). The CMs used were from A549 cells previously incubated for 48 h with vehicle (Veh) or the indicated concentrations of the test compounds. All percentage values given refer to serum-free DMEM (unconditioned medium, UCM) set to 100%. Data represent mean ± SEM of *n* = 6 ((**A**,**B**,**E**), migration and tube formation), *n* = 3–4 (**C**,**D**), or *n* = 8 ((**E**), viability) per group. ** *p* ≤ 0.01, *** *p* ≤ 0.001 vs. UCM; # *p* ≤ 0.05, ## *p* ≤ 0.01, ### *p* ≤ 0.001 vs. CM of vehicle-treated A549 cells; one-way ANOVA with Bonferroni post hoc test.

To investigate a possible indirect antiangiogenic effect of JZL184 and 2-AG by modulating the microenvironment of tumor cells, an alternative experimental setup was next used. For this purpose, CM from A549 cells treated with vehicle, JZL184, or 2-AG (both at concentrations between 0.1 and 10 µM) were tested for their effect on the angiogenic properties of HUVECs. To this end, HUVECs were suspended in CM of appropriately treated A549 cells and seeded in transwell chambers for migration analysis, in 48-well plates for tube formation assessment, or in 96-well plates for viability quantification, followed by incubation for 24 h (migration, viability) or 2 h (tube formation). As shown in Figure 2 (all panels), CM of A549 cells incubated with vehicle for 48 h increased migration, viability, and tube formation of HUVECs compared with HUVECs suspended in serum-free DMEM (unconditioned medium, UCM). CM of A549 cells treated with JZL184 (Figure 2A) and 2-AG (Figure 2B) showed concentration-dependent inhibition of migration and tube formation of HUVECs compared with CM of A549 cells treated with vehicle, whereas CM-induced viability of HUVECs was not significantly altered by either test substance. Regarding the strong effect of JZL184 concentrations of 0.1, 1, and 10 µM in increasing the endocannabinoid 2-AG in appropriately treated A549 cells, we refer to a previous work of our group [23].

### 3.2. The MAGL Inhibitors JW651 and MJN110 also Indirectly Inhibit the Angiogenic Capabilities of HUVECs, Whereas Inhibition of Endogenous 2-AG Synthesis Leads to Enhanced Tube Formation

To rule out a nonspecific effect of JZL184 on the inhibition of angiogenic properties of HUVECs exposed to CM from appropriately treated A549 cells, we further investigated whether other MAGL inhibitors such as JW651 and MJN110 also inhibit migration and tube formation of HUVECs. As a result of these experiments, a concentration-dependent inhibition of HUVEC migration and tube formation by CM of A549 cells treated with JW651 (Figure 2C) and MJN110 (Figure 2D) was demonstrated, whereas HUVEC viability remained virtually unchanged under these conditions. 

To further elucidate the role of endogenous 2-AG as a modulator of the angiogenic properties of endothelial cells in the context of tumor–endothelial interaction, it was then investigated whether inhibition of 2-AG synthesis by blocking diacylglycerol lipase (DAGL), the major enzyme for 2-AG synthesis [33], leads to the promotion of angiogenesis. For this purpose, the DAGL inhibitor RHC 80267 was used. Indeed, CM of A549 cells treated with RHC 80267 was found to increase HUVEC tube formation, which was statistically significant at a final concentration of 1 µM (Figure 2E). However, HUVEC migration and viability remained virtually unchanged. 

### 3.3. Activation of CB_1_ Receptors, but Not Reduction in Free Fatty Acids, Mediates the Antiangiogenic Properties of CM from A549 Cells Treated with JZL184 on HUVECs

**Figure 3:**
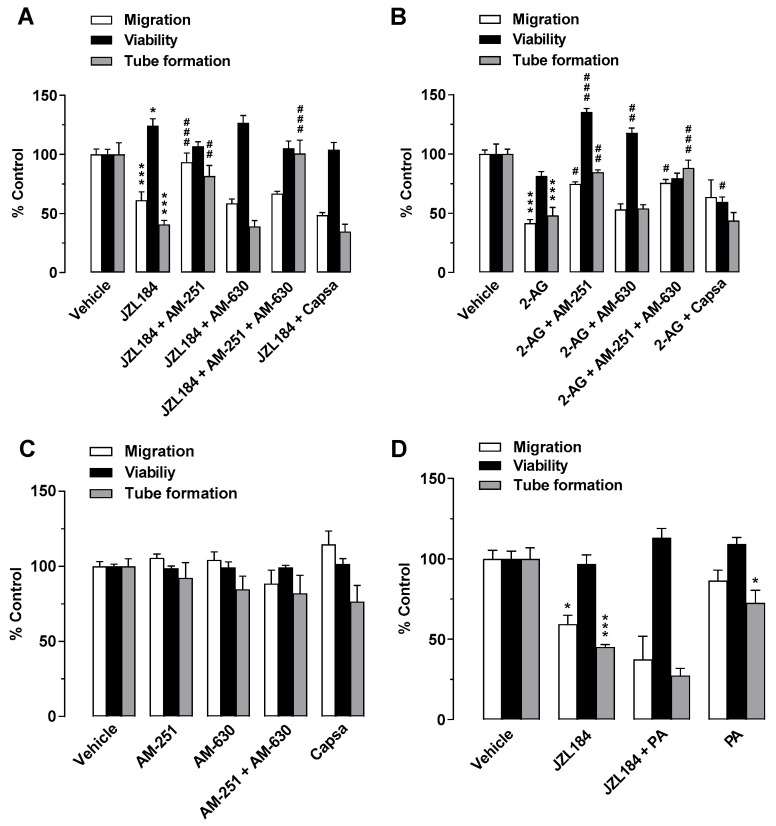
Evaluation of the role of the endocannabinoid system in the antiangiogenic effect of JZL184 (**A**) and 2-AG (**B**) on HUVECs by testing the effect of AM-251 (CB_1_ antagonist), AM-630 (CB_2_ antagonist), and capsazepine (Capsa, TRPV1 antagonist) on the antiangiogenic effect of CM from A549 cells treated with 1 µM JZL184 (**A**) or 2-AG (**B**). A549 cells were pre-incubated with the respective receptor antagonist (all tested at a final concentration of 1 µM) for 1 h and then incubated with vehicle, JZL184, or 2-AG for 48 h before CM was collected. Migration (Boyden chamber assay, white bars), viability (WST-1 assay, black bars), and tube formation (tube formation assays, gray bars) of HUVECs were determined after incubation with CM from A549 cells for 24 h (migration and viability assay) or 2 h (tube formation analysis). The control experiment shown in (**C**) demonstrates the effect of CM of A549 cells previously incubated with AM-251, AM-630, and capsazepine alone for 48 h on HUVEC migration, viability, and tube formation. In the experiment shown in (**D**), modulation of angiogenic properties by CM of A549 cells previously treated for 48 h with vehicle, JZL184 (1 µM), palmitic acid (PA, 10 µM), or the combination of JZL184 (1 µM) and PA (10 µM) was determined. PA was used to supplement potentially reduced free fatty acids due to MAGL inhibition. All percentage values given refer to HUVECs suspended in vehicle-containing CM set to 100%. Data represent mean ± SEM of *n* = 9 (**A**), *n* = 3 ((**B**,**D**), tube formation), *n* = 6 ((**C**), migration, tube formation; (**D**), migration), *n* = 7 ((**C**), viability), or *n* = 4 ((**D**), viability) per group. * *p* ≤ 0.05, *** *p* ≤ 0.001 vs. CM of vehicle-treated A549 cells; # *p* ≤ 0.05, ## *p* ≤ 0.01, ### *p* ≤ 0.001 vs. CM of JZL184- or 2-AG-treated A549 cells; one-way ANOVA with Bonferroni (**A**,**B**,**D**) or Dunnett (**C**) post hoc test.

To investigate a possible role of the cannabinoid receptors CB_1_ and CB_2_ and transient receptor potential vanilloid 1 (TRPV1) in mediating the antiangiogenic effects of JZL184 and 2-AG, the influence of antagonists against CB_1_ (AM-251), CB_2_ (AM-630), and TRPV1 (capsazepine) was then examined. Receptor antagonists were used at a concentration of 1 µM, which were reported to inhibit CB_1_, CB_2_, and TRPV1 activation [6,23,34,35,36]. As shown in Figure 3A, the antimigratory and anti-tube-forming effects on HUVECs were significantly suppressed in the presence of CM from JZL184-treated A549 cells when the cancer cells were pretreated with the CB_1_ antagonist AM-251. In contrast, both CB_2_ antagonist and TRPV1 antagonist were inactive in this regard. Moreover, the combination of CB_1_ and CB_2_ antagonists decreased the tube formation inhibitory activity of JZL184. However, a corresponding inhibitory effect on migration was not observed. In the case of 2-AG, the migration- and tube-forming inhibitory effects of this agent were significantly abolished by the CB_1_ antagonist AM-251 and by the combination of AM-251 and AM-630 (Figure 3B). CM from A549 cells treated with receptor antagonists in the absence of JZL184 and 2-AG had no significant effect on the angiogenic capacities of HUVECs (Figure 3C). 

In addition to the increase in 2-AG levels, decreased synthesis of protumorigenic fatty acids were described as a consequence of MAGL inhibition [21]. To rule out a possible decrease in protumorigenic free fatty acids as a cause for the observed antiangiogenic effects of JZL184, further migration, viability, and tube formation assays were performed using palmitic acid with the aim of add-back. However, the addition of palmitic acid did not reverse the antimigratory effect or tube formation inhibitory activity of CM from JZL184-treated A549 cells (Figure 3D).

### 3.4. JZL184 and 2-AG Increase the Release of TIMP-1 from A549 Cells

**Figure 4:**
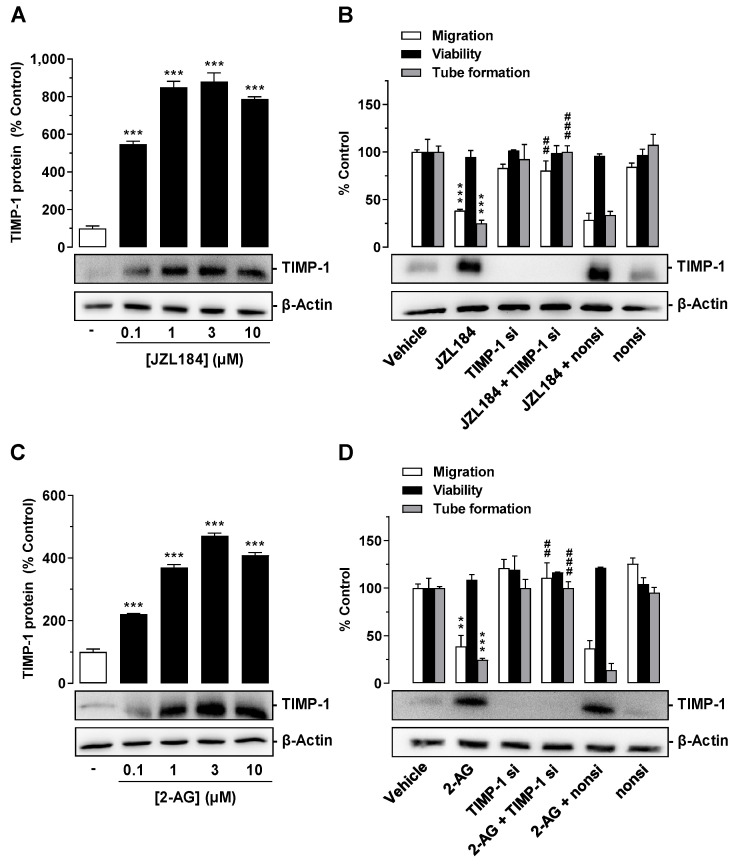
Effect of JZL184 (**A**,**B**) and 2-AG (**C**,**D**) on TIMP-1 protein expression in A549 cells and angiogenic abilities of HUVECs suspended in conditioned medium (CM) of vehicle- or JZL184-treated A549 cells in the presence or absence of TIMP-1 siRNA. In (**A**,**C**), the concentration-dependent effects of JZL184 (**A**) and 2-AG (**C**) on TIMP-1 protein expression are shown. A549 cells were incubated with vehicle and the respective concentration of JZL184 or 2-AG for 48 h followed by Western blot analysis. All percentage values given refer to vehicle-treated cells set to 100%. Data (**A**,**C**) represent mean ± SEM obtained from densitometric analysis of *n* = 4 experiments per group. In (**B**,**D**), A549 cells were incubated with transfection reagent in the absence of any siRNA (first and second triplets or bands) and transfected with TIMP-1 siRNA (TIMP-1 si; third and fourth triplets or bands) or non-silencing siRNA (nonsi; fifth and sixth triplet or bands) for 24 h in 10% FCS containing DMEM. Thereafter, A549 cells were washed and treated with vehicle, 1 μM JZL184 (**C**), or 1 µM 2-AG (**D**) for 48 h in serum-free DMEM prior to collection of CM. Migration (Boyden chamber assay, white bars), viability (WST-1 test, black bars), and tube formation (tube formation assays, gray bars) of HUVECs was measured after suspension in CM from A549-treated cells. The incubation time of HUVECs was 24 h for migration and viability testing and 2 h for tube formation analysis. Monitoring of TIMP-1 protein was performed in parallel using CM obtained from A549 cells. The Western blot images shown here are representative of a total of three (**B**) or four (**D**) experiments performed. For analysis of angiogenesis data in (**B**,**D**), vehicle-containing CM was set at 100%. Data represent mean ± SEM of *n* = 3 ((**B**), migration, tube formation; (**D**)) or *n* = 4 ((**B**), viability) per group. ** *p* ≤ 0.01, *** *p* ≤ 0.001 vs. corresponding vehicle control; ## *p* ≤ 0.01, ### *p* ≤ 0.001 vs. the respective JZL184 (**B**) or 2-AG (**D**) group without siRNA; one-way ANOVA with Dunnett (**A**,**C**) or Bonferroni (**B**,**D**) post hoc test.

TIMP-1 was shown to be a key factor in the antiangiogenic effects of various cannabinoids of plant and synthetic origin [6]. Since TIMP-1 induction in A549 and H358 cells was recently described for JZL184 and 2-AG [23], the involvement of this factor in the antiangiogenic response of both compounds was next investigated. For this purpose, A549 cells were treated with JZL184 or 2-AG at a concentration range between 0.1 and 10 µM. After a 48 h incubation period, cell culture media were collected from A549 cells and analyzed for modulation of TIMP-1 expression by Western blot. Supernatant evaluation was supplemented with analyses of cell lysates for detection of β-actin expression to rule out toxic effects of the test compounds. Under these conditions, as shown in Figure 4, significant TIMP-1 upregulation was detected in the cell culture media in response to treatment with all concentrations of JZL184 (Figure 4A) or 2-AG (Figure 4C) tested, with a plateau beginning at concentrations of 1 µM (JZL184) or 3 µM (2-AG). 

### 3.5. TIMP-1 Released from A549 Cells Mediates the Antiangiogenic Effect of JZL184 and 2-AG on HUVECs

To investigate a causal relationship between JZL184- and 2-AG-mediated TIMP-1 induction in A549 cells and the associated reduced angiogenic abilities in HUVECs, a specific siRNA targeting TIMP-1 was tested for its influence on JZL184- or 2-AG-induced inhibition of migration and tube formation. As shown in Figure 4B, the knockdown of JZL184-induced TIMP-1 expression in A549 cells led to a significant inhibition of the antimigratory and anti-tube-forming effects of CM derived from JZL184-treated A549 cells. On the other hand, viability remained unaffected. Western blot experiments showed a highly effective knockdown of induced and basal TIMP-1 expression. Since the expression of the target protein in TIMP-1 siRNA transfected cells was no longer detectable under these circumstances, densitometric quantification of TIMP-1 was not applied in these experiments (Figure 4B,D). The non-silencing siRNA left the JZL184-induced TIMP-1 induction and the antimigratory and anti-tube-forming properties of JZL184 virtually unchanged. Similar results were obtained in experiments with 2-AG (Figure 4D).

### 3.6. CM from JZL184- or 2-AG-Treated H358 Cells, Another Lung Cancer Cell Line, but Not from the Non-Cancerous Bronchial Epithelial Cell Line BEAS-2B Mediate Antiangiogenic Effects on HUVECs

**Figure 5:**
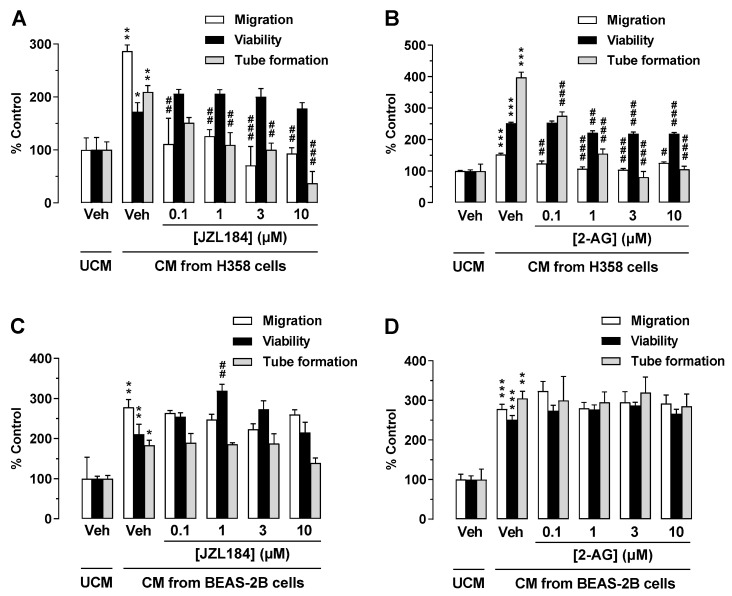
Effect of conditioned medium (CM) derived from another lung cancer cell line (H358) and from a non-cancerous bronchial epithelial cell line (BEAS-2B) on the angiogenic capacities of HUVECs. Migration (Boyden chamber assay, white bars), viability (WST-1 assay, black bars), and tube formation (tube formation assays, gray bars) of HUVECs were determined after suspension in CM from vehicle-, JZL184-, and 2-AG-treated H358 (**A**,**B**) or BEAS-2B cells (**C**,**D**). To generate CM, H358 or BEAS-2B cells were incubated for 48 h with vehicle (Veh) or the indicated concentrations of test compounds. The exposure time of HUVECs to the indicated CM was 24 h for the migration and viability assay and 2 h for tube formation analysis. Vehicle-containing UCM was set at 100%. Data represent mean ± SEM of *n* = 3 ((**A**–**D**), tube formation; (**A**–**C**), migration; (**A**), viability), *n* = 4 ((**D**), migration), *n* = 5 ((**B**), viability), or *n* = 6 ((**C**), viability) per group. * *p* ≤ 0.05, ** *p* ≤ 0.01, *** *p* ≤ 0.001 vs. UCM; # *p* ≤ 0.05, ## *p* ≤ 0.01, ### *p* ≤ 0.001 vs. CM of vehicle-treated A549 cells; one-way ANOVA with Bonferroni post hoc test.

To exclude that the proven antiangiogenic effects are limited to CM of A549 cells, key experiments were performed with H358 cells, another lung cancer cell line. As shown in Figure 5A,B, CM from H358 cells incubated with JZL184 or 2-AG exhibited a similar pattern of effects on the angiogenic properties of endothelial cells as CM from A549 cells treated in the same manner. Interestingly, in the case of the experiment shown in Figure 5B, a significant inhibition of viability was also registered, although of a comparatively small magnitude. In contrast, and in accordance with the hypothesis that this antiangiogenic cannabinoid effect is strictly limited to malignant cells, CM from JZL184- or 2-AG-treated BEAS-2B cells, a normal bronchial epithelial cell line [37,38], did not inhibit migration, tube formation, or viability of HUVECs when compared to CM of vehicle-treated cells (Figure 5C,D).

### 3.7. TIMP-1 Is Also the Mediator of the Antiangiogenic Effect of CM from JZL184- and 2-AG-Treated H358 Cells on HUVECs

**Figure 6:**
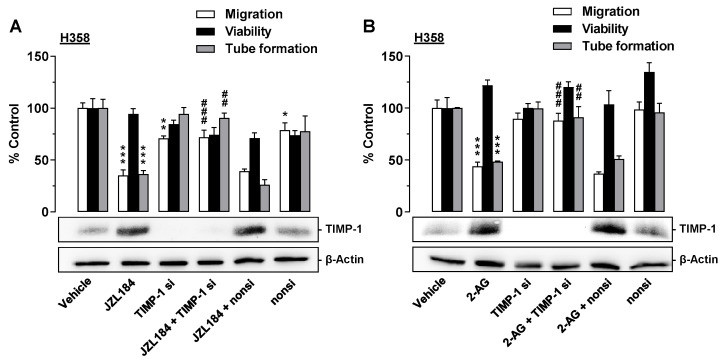
Angiogenic capabilities of HUVECs suspended in conditioned medium (CM) from vehicle- or JZL184-treated H358 cells in the presence or absence of TIMP-1 siRNA. H358 cells were incubated with transfection reagent in the absence of any siRNA (first and second triplets or bands) and transfected with TIMP-1 siRNA (TIMP-1 si; third and fourth triplets or bands) or non-silencing siRNA (nonsi, fifth and sixth triplet or bands) for 24 h in 10% FCS containing DMEM. Thereafter, H358 cells were washed and treated with vehicle, 1 μM JZL184 (**A**), or 1 µM 2-AG (**B**) for 48 h in serum-free DMEM prior to collection of CM. Migration (Boyden chamber assay, white bars), viability (WST-1 test, black bars), and tube formation (tube formation assays, gray bars) of HUVECs was measured after suspension in CM from H358-treated cells. The incubation time of HUVECs was 24 h for migration and viability testing and 2 h for tube formation analysis. Monitoring of TIMP-1 protein was performed in parallel using CM from H358 cells. The Western blot images shown here are representative of a total of four experiments performed in each case. For analysis of angiogenesis data, vehicle-containing CM was set at 100%. Data represent mean ± SEM of *n* = 6 ((**A**), migration; (**B**), migration), *n* = 3 ((**A**), viability, tube formation; (**B**), tube formation), or *n* = 4 ((**B**), viability) per group. * *p* ≤ 0.05, ** *p* ≤ 0.01, *** *p* ≤ 0.001 vs. corresponding vehicle control; ## *p* ≤ 0.01, ### *p* ≤ 0.001 vs. the respective JZL184 or 2-AG group without siRNA; one-way ANOVA with Bonferroni post hoc test.

To demonstrate a causal relationship between TIMP-1 induction in JZL184- or 2-AG-treated H358 cells and the antiangiogenic effect of the corresponding CM on HUVECs, siRNA experiments were also performed here. Thereby, transfection of the cells with TIMP-1 siRNA led to a complete knockdown of TIMP-1 induction induced by JZL184 (Figure 6A) or 2-AG (Figure 6B) as well as basal TIMP-1 formation. In the case of both JZL184 and 2-AG, the knockdown was accompanied by significant inhibition of the antimigratory and anti-tube-forming effects of the CM derived from the correspondingly treated H358 cells. In contrast, the non-silencing siRNA used as negative control left the antiangiogenic properties of the CM as well as the basal and JZL184- or 2-AG-induced TIMP-1 formation virtually unchanged. The viability of HUVECs was not significantly changed between groups.

### 3.8. The MAGL Inhibitor JZL184 Inhibits Tumor Growth in Nude Mice Accompanied by a Reduction in the Angiogenesis Marker CD31 and an Upregulation of the Antiangiogenic Mediator TIMP-1 in the Corresponding Xenografts

**Figure 7:**
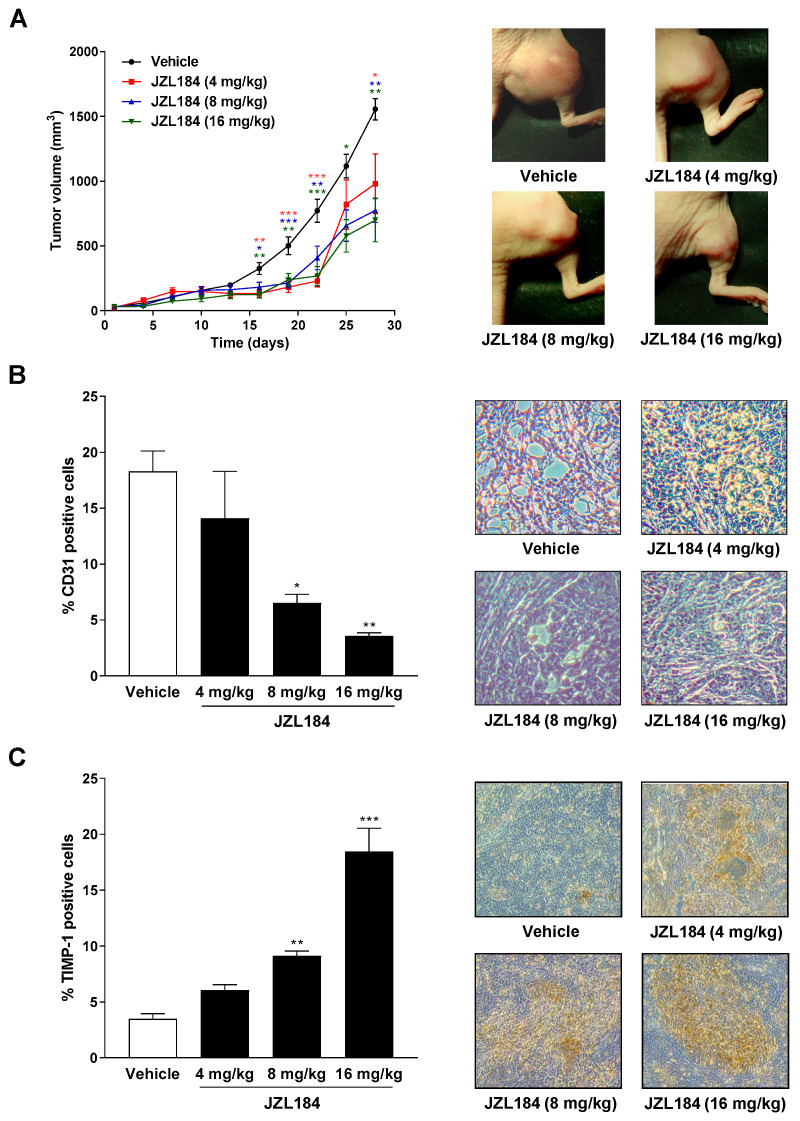
Impact of MAGL inhibitor JZL184 on the growth of A549 xenografts from nude mice as well as on the number of CD31- (angiogenesis and vascularization marker) and TIMP-1-positive cells in xenografts. Tumors were generated by subcutaneous inoculation of 1 × 10^7^ A549 cells into the right dorsal flank. Animals were treated with either vehicle or JZL184 (4, 8, or 16 mg/kg i.p.) every 72 h for 28 days. Tumor size was measured with an external caliper and calculated as described in Materials and Methods and are shown as tumor volumes over time in panel **A**. The images beside the time-course were taken from representative tumors on day 28. Quantification of CD31 (**B**) and TIMP-1 (**C**) was performed by counting the positively stained cells of xenograft-derived paraffine sections stained with the indicated antibodies and calculating the percentage relative to the total number of cells per field of view. Values are means ± SEM of *n* = 7–8 (**A**), *n* = 4 (**B**), or *n* = 5 (**C**) animals per group. * *p* ≤ 0.05, ** *p* ≤ 0.01, *** *p* ≤ 0.001 vs. vehicle; one-way ANOVA with Dunnett post hoc test.

To assess the influence of the MAGL inhibitor JZL184 on the growth and angiogenesis of lung tumor cells in vivo, athymic nude mice were xenografted with A549 cells and treated with vehicle or JZL184 (4, 8, or 16 mg/kg, every 72 h) for four weeks. As shown in Figure 7A, JZL184 caused profound decrease in tumor volume. Interestingly, a dose dependence only became apparent at the last two measurement time points. The tumor-regressive effect of JZL184 was accompanied by a dose-dependent reduction in the angiogenesis marker CD31 (Figure 7B) and a likewise dose-dependent increase in TIMP-1-positive cells in xenografts. Both the downregulation of CD31 and the upregulation of TIMP-1 were significant starting at a dose of 8 mg/kg JZL184.

## 4. Discussion

The present study provides evidence for an antiangiogenic effect of the MAGL inhibitor JZL184 in vitro, which was associated with a pronounced dose-dependent reduction in lung cancer growth in vivo. The mechanistic background of reduced tumor angiogenesis by JZL184 in vitro is an elevation of the endocannabinoid 2-AG in cancer cells. Here, the 2-AG increase results from the inhibition of MAGL and leads to a reduction in the angiogenic capacities of endothelial cells in the tumor microenvironment via a CB_1_-dependent pathway, with increased TIMP-1 release from lung tumor cells being causative. A tumor-regressive effect of JZL184 in association with a decrease in an angiogenesis marker was finally also confirmed in vivo. 

There are several results supporting this notion. First, all CM derived from A549 lung cancer cells treated with the three different MAGL inhibitors JZL184, JW651, and MJN110 inhibited the migration and tube formation of HUVECs, thus ruling out an off-target effect of JZL184, which was in the focus of subsequent more detailed mechanistic studies. Secondly, CM of A549 treated with the MAGL substrate 2-AG mimicked this antiangiogenic effect. Third, the in vitro results regarding the antiangiogenic effect of JZL184 and 2-AG were confirmed at the in vivo level by demonstrating a tumor-regressive effect of JZL184 in association with a decrease in CD31-positive cells in xenografts from appropriately treated nude mice. Fourth, the inhibitory effect of CM from cancer cells treated with JZL184 or 2-AG on tube formation and endothelial cell migration potential was reduced by AM-251, a CB_1_ receptor antagonist. In contrast, receptor antagonists against CB_2_ and TRPV1, as well as the restoration of fatty acid stores through the addition of palmitic acid, left the antiangiogenic effect of JZL184 largely unaffected. Fifthly and finally, a knockdown of JZL184- and 2-AG-induced TIMP-1 expression by specific siRNA in both A549 and H358 cells inhibited the antimigratory and anti-tube-forming properties of the corresponding cancer cell CM on HUVECs, underlining the crucial role of TIMP-1 in this antiangiogenic mechanism. Moreover, increases in TIMP-1 protein could also be registered in vivo in A549 xenografts from nude mice treated with JZL184.

The involvement of the CB_1_ receptor in the anticancer effects of JZL184 and 2-AG, as shown by us for the antiangiogenic action, is supported by results from our own and other groups. Accordingly, an at least partial involvement of the CB_1_ receptor was reported for the growth-inhibiting effect of JZL184 on prostate cancer cells [22]. Furthermore, there is evidence in the literature for a CB_1_-dependent anti-invasive effect of 2-AG on prostate cancer cells, which likewise supports the data collected in our work [39]. In addition, another recent study of our group pointed out that the antimetastatic and anti-invasive effects of JZL184 in A549 cells are mediated by a CB_1_ receptor-dependent signaling pathway [23]. The latter investigation confirmed the efficacy of JZL184 as a MAGL inhibitor in terms of its selective 2-AG enhancing effect in A549 cells. Thereby, a significant increase in intracellular 2-AG levels was shown after treatment with JZL184 at concentrations ranging from 0.01 µM to 10 µM [23]. In contrast, no increases were observed for the two fatty acid amide hydrolase (FAAH) substrates oleoylethanolamide and palmitoylethanolamide, and concentrations of the major FAAH substrate anandamide (*N*-arachidonoylethanolamine) were below the limit of quantification [23]. 

Although the CB_2_ receptor antagonist AM-630 had no significant effect on restoring JZL184- and 2-AG-induced inhibition of endothelial migration and tube formation, a previous study suggests that this approach is efficient in terms of CB_2_ receptor blockade. Thus, in a comparable experimental setup, AM-630 significantly abrogated the antimigratory effect on HUVECs induced by CM of cannabidiol- or Δ^9^-tetrahydrocannabinol-treated A549 cells [6]. Similarly, significant inhibition of migration and tube formation was demonstrated for CM of A549 cells previously incubated with the selective CB_2_ agonist JWH-133, which was also confirmed in the case of migration in pilot experiments for lung tumor cell lines H358 and H460 [6]. Taken together, these data suggest that angiogenesis of HUVECs can in principle be inhibited by the CM of cancer cells treated with a CB_2_ receptor agonist. On the other hand, the CB_2_ receptor does not play a critical role in the antiangiogenic effect induced by CM of JZL184- and 2-AG-treated lung cancer cells.

While this was ruled out in the present study by add-back experiments with palmitic acid, it remains to be noted that, in addition to the upregulation of 2-AG, the reduction in free fatty acids may also be involved in the effects of MAGL inhibitors. Accordingly, one study showed that the MAGL inhibitor JZL184 blocked oleic acid-stimulated proliferation of glioblastoma cells via modulation of triglyceride metabolism [40]. Consistent with this, a reduction in free fatty acids as a result of MAGL inhibition was described as the main cause of the anticancer potential of MAGL inhibition in breast, ovarian, and melanoma cancer cells [21]. These results were confirmed by in vivo experiments in which MAGL was knocked down by a small hairpin RNA, resulting in a decrease in tumor growth that was completely counter-regulated in mice fed a high-fat diet.

It is remarkable that RHC 80267 as a DAGL inhibitor, which accordingly causes an inhibition of 2-AG synthesis in tumor cells [24], only induced tube formation, while in contrast to our expectations, the migration of HUVECs remained unchanged. In the experiments of another group, RHC 80267 increased the invasion of the androgen-independent prostate cancer cell lines PC-3 and DU-145 [24], but showed no effect on the invasion of androgen-sensitive LNCaP prostate cancer cells. In addition, RHC 80267 was reported to reduce the motility of N-cadherin-expressing cell lines, while having no effect on N-cadherin negative cells [41]. Finally, RHC 80267 was found to decrease rather than increase the cellular viability of human colon adenocarcinoma Colo-205 cells [42]. Considering that RHC 80267 also strongly affects arachidonic acid levels and diacylglycerol-dependent intracellular signaling by inhibiting DAGL, it becomes clear that the action of such substances is associated with further complex intracellular events in addition to their influence on the endocannabinoid system. The cause of the lack of effect of RHC 80267 on the migration of HUVECs should be considered accordingly in further investigations.

The involvement of TIMP-1 in the antiangiogenic activity of JZL184 and 2-AG is consistent with a number of other studies that showed TIMP-1 to be an angiogenesis inhibitor [5,6,7,8,9,43,44]. In addition, the current data are in line with a previous report by our group that showed several cannabinoid compounds to mediate antiangiogenic activity via increased TIMP-1 release from A549 cells [6]. A similar mechanism was also recently verified for the antiangiogenic effect of the TIMP-1-inducing chemotherapeutic agent cisplatin [5]. The fact that JZL184 and 2-AG mediated a TIMP-1-dependent antiangiogenic effect without reducing the viability of HUVECs is consistent with previous observations that also found the proliferative activity of endothelial cells to be virtually unchanged by exogenous TIMP-1 [6,45]. The exact mechanism by which TIMP-1 released by lung cancer cells causes inhibition of migration and tube formation of HUVECs requires further investigation. An inhibition of MMP-2 [46] or an inhibition of MMP-9, for whose knockdown a cellular switch from a migratory to a stationary phase via a Paxillin/RhoA-dependent reorganization of the cytoskeleton and a stabilization of the β-catenin/E-cadherin complex was shown, could be possible [47]. Moreover, effects induced by TIMP-1 independently of its proteolysis-modifying action are also conceivable [48,49,50,51]. Consistent with this, several groups indeed detected changes in migration triggered by modulation of the MMP/TIMP system, even when using uncoated inserts, the passage of which does not require proteolytic action [47,52,53].

In contrast to the results reported here, other studies found that cannabinoids directly inhibit the angiogenic capacity of HUVECs [12,14,30,54,55]. However, the results from these studies only partially contradict our findings due to different experimental concepts, higher concentrations, or other cannabinoid compounds used in the respective experiments. The tendency of cannabinoids to have enhancing rather than inhibitory effects on the angiogenic capacity of endothelial cells upon direct exposure, presented here and in a previous study conducted by our group [6], is consistent with several other studies suggesting that cannabinoids support vascular integrity [56,57,58]. In this context, it should be noted that the concept of indirect antiangiogenic cannabinoid action, which presupposes the presence of an intermediate cell type, is also supported by experiments of other authors. Thus, only a change in the microenvironment of activated mastocytes by the endocannabinoid-like substance palmitoylethanolamide led to a reduction in HUVEC proliferation [59]. Our data also suggest that the antiangiogenic effect of the test substances is limited to the presence of cancer cells in the endothelial microenvironment, since CM of JZL184- or 2-AG-treated normal bronchial epithelial cells did not induce inhibition of migration and tube formation of HUVECs. Transferred to in vivo conditions, this would possibly mean that the antiangiogenic effect is limited to the tumor only and that the corresponding test substances exert less undesirable effects on other tissues.

Collectively, the data presented in the present work are in line with a body of experimental evidence in recent years that suggests that inhibition of MAGL may be a promising pharmacotherapeutic strategy for the treatment of cancer. Regarding the demonstrated growth inhibitory effect of JZL184 on A549 xenografts, our results are in good agreement with data from other authors who reported the tumor-regressive properties of JZL184 in a mouse xenograft model with MAGL-overexpressing hepatocellular cancer cells [25] or in a colon cancer cell xenograft model [24]. It can be suggested that the antiangiogenic effect of JZL184 plays an important role in its tumor-regressive effect shown in the present study. Indeed, in a recent investigation by our group, JZL184 had virtually no effect on the proliferation and viability of A549 lung tumor cells [23]. Here, JZL184 showed no significant effect in colony formation assay when A549 cells were incubated with MAGL inhibitor for two weeks. Similarly, the viability and proliferation of A549 cells was not significantly reduced up to a JZL184 concentration of 10 µM at a treatment duration of 48 h [23]. However, it is also worth noting that a recent publication found that inhibition of MAGL promoted rather than inhibited cancer progression in mice [60]. The authors obtained their results in MAGL knockout mice, which had a higher incidence of neoplasia in multiple organs with splenomegaly and particularly promoting effects on the progression of lung cancer. Thereby, MAGL deficiency in this model was associated with increased epidermal growth factor receptor expression and phosphorylation. On the other hand, numerous other preclinical studies suggest that MAGL inhibitors affect various stages of carcinogenesis (for review, see [19,20]). Moreover, inhibition of MAGL was shown to reduce cachexia in a mouse model of bone cancer [26] and lithium chloride-induced vomiting in shrews [61], and to abolish chemotherapy-induced neuropathy in mice [29], which should be considered as useful concomitant effects in further evaluation of MAGL inhibitors as potential cancer therapeutics.

## 5. Conclusions

The present study provides a first-time proof for the antiangiogenic action of the MAGL inhibitor JZL184 that was associated with a profound growth reduction of lung cancer xenografts. Detailed in vitro analyses showed that JZL184 increased the release of TIMP-1 from lung cancer cells, suppressing vascularization in the cancer environment. In this way, undesirable side effects on healthy tissue could be avoided. Although meaningful clinical studies on the efficacy and safety of MAGL inhibitors in cancer treatment are still pending, the preclinical data presented here give rise to hope that such a strategy could significantly enrich pharmacotherapies for tumor diseases.

## Data Availability

Data are available upon reasonable request from the first author.
